# Advanced gelatin-based vascularization bioinks for extrusion-based bioprinting of vascularized bone equivalents

**DOI:** 10.1038/s41598-020-62166-w

**Published:** 2020-03-24

**Authors:** A. Leucht, A.-C. Volz, J. Rogal, K. Borchers, P. J. Kluger

**Affiliations:** 1 Institute of Interfacial Process Engineering and Plasmatechnology IGVP, University of Stuttgart, Stuttgart, Germany; 2 Fraunhofer Institute for Interfacial Engineering and Biotechnology IGB, Stuttgart, Germany; 3 Reutlingen Research Institute, Reutlingen University, Reutlingen, Germany; 4 RWTH Aachen University, Aachen, Germany

**Keywords:** Biomaterials, Bone development, Differentiation, Biomedical engineering, Biomaterials

## Abstract

Bone tissue is highly vascularized. The crosstalk of vascular and osteogenic cells is not only responsible for the formation of the strongly divergent tissue types but also for their physiological maintenance and repair. Extrusion-based bioprinting presents a promising fabrication method for bone replacement. It allows for the production of large-volume constructs, which can be tailored to individual tissue defect geometries. In this study, we used the all-gelatin-based toolbox of methacryl-modified gelatin (GM), non-modified gelatin (G) and acetylated GM (GMA) to tailor both the properties of the bioink towards improved printability, and the properties of the crosslinked hydrogel towards enhanced support of vascular network formation by simple blending. The vasculogenic behavior of human dermal microvascular endothelial cells (HDMECs) and human adipose-derived stem cells (ASCs) was evaluated in the different hydrogel formulations for 14 days. Co-culture constructs including a vascular component and an osteogenic component (i.e. a bone bioink based on GM, hydroxyapatite and ASCs) were fabricated via extrusion-based bioprinting. Bioprinted co-culture constructs exhibited functional tissue-specific cells whose interplay positively affected the formation and maintenance of vascular-like structures. The setup further enabled the deposition of bone matrix associated proteins like collagen type I, fibronectin and alkaline phosphatase within the 30-day culture.

## Introduction

In bone tissue engineering (BTE), osteogenic cells, extracellular matrix (ECM) components and various stimuli are combined to build constructs that resemble native bone tissue (BT) in structure and function^1^. Such constructs could be applied to treat *in vivo* bone defects^2^. Bone is a highly vascularized organ^3^. A high degree of vascularization is essential in engineered tissue constructs to supply all cellular components with sufficient nutrients during culture and after implantation to avoid necrotic cores of large-scale constructs. Current *in vivo* vascularization strategies range from smart scaffolds, which are made of porous biomaterials with bioactive molecules like angiogenic growth factors, to prevascularized implants, where grafts are first implanted into a well-vascularized body site, removed upon sufficient vascularization and subsequently placed at the actual site of defect^4–10^. *In vitro* vascularization is another approach, in which endothelial cells (ECs) are used with supporting factors, like angiogenic growth factors or additional cell types and supporting ECM components, to facilitate the *de novo* generation of vascular-like structures^11^. Accelerated vessel formation and improved maintenance of the formed structures were shown before in co-culture with supporting cells like mesenchymal stem cells (MSCs)^12,13^. Here, MSCs are assumed to function as perivascular cells, supporting angiogenic processes like vessel sprouting, elongation and the stabilization of built vessels^14^. Furthermore, processes like angiogenesis and osteogenesis are interdependent processes^15^. While ECs respond to vascular endothelial growth factor (VEGF) released by osteoblasts with migration and proliferation, osteogenic differentiation is vice versa supported by bone morphogenetic protein-2 and -4, released by ECs^16^. Thus, the integration of a vascular system is not only important for the general supply of the cells, but as well for the development of bone-specific cell types.

While regular TE approaches only partly manage to reconstruct targeted geometries, the emerging method of bioprinting could help to set up anatomic features of native bone *in vitro*^17–21^. Bioprinting may serve to construct hierarchical scaffold geometries that support distribution of nutrients and oxygen. Kang *et al*. e.g. used 3D bioprinting to generate scaffolds with microchannels out of stable polycaprolacton struts and a hydrogel filling for calvarial bone reconstruction^22^. Alternatively, alternate deposition of bioinks containing different cell-types, such as stem cells and ECs, and cell-type specific hydrogel-formulations is used to produce pre-structured tissue models^15^. Such approaches serve to investigate if maturation of functional tissue can be achieved by self-organization, i.e. formation of capillary networks.

Within additive manufacturing, BTE is most often based on extrusion-based bioprinting, whereby strands of a bioink consisting of an extrudable biomaterial and tissue-specific cells are placed layer-wise on a substrate using a nozzle and pneumatic or mechanical forces^23^. Extrusion-based bioprinting offers the possibility to print bioinks of high viscosity, including high cell numbers, within a relatively short time^15^. Independent of the targeted tissue, bioinks have to fulfill two general requirements: First, they should show high cell compatibility and second, the physical properties of the material should meet the requirements of extrusion-based bioprinting, which requires viscosities in a range of approx. 30 mPa s to 6 × 10^7^ mPa s^24^. Therefore, they should exhibit sufficient viscosity to allow for the extrusion of bioinks with minimal shear stress for the included cells and form stable tissue constructs during culture or ideally even after implantation. Hydrogels can mimic the natural ECM environment of the cells through their high water-binding capacity. Natural ECM-derived polymers like collagen or gelatin are very suitable for TE as they exhibit enzymatic cleavage sites, which e.g. allow ECs to partly degrade the scaffold and migrate^25^. In contrast to alginate or fibrinogen, ECM proteins provide cell anchorage sites that enable MSC and EC adhesion and spreading^26^. These characteristics are indispensably needed to facilitate fundamental steps of angiogenesis, like vessel elongation or lumen formation^14^.

Gelatin is soluble in warm water and forms physical hydrogels at low temperatures. Thus, below the melting temperature non-modified gelatin (G) can be used to increase the viscosity of bioinks for extrusion-based printing. Physically gelled gelatin can temporarily stabilize hydrogel-based scaffolds during the printing process^27^. However, gelatin hydrogels melt and solubilize at physiological 37 °C and therefore have to be chemically crosslinked to form stable hydrogel scaffolds for TE. Modification of gelatin with methacryl-functions provides gelatin methacryloyl (GM), i.e. ECM-derived biopolymers which can be chemically crosslinked by radical-induced reactions^28,29^. Currently, various bioink formulations based on GM are investigated to identify bioinks that support specific cell-types to reconstruct functional tissue^30–33^. Hydroxyapatite (HAp), which makes up about 60% of BT in the human body, and tricalcium phosphate have been confirmed as osteogenic differentiation-supporting materials^34–37^. While the pure use of such materials is not applicable in extrusion-based bioprinting, formulations of mineral components with GM hydrogels can be used to yield bioinks that support BTE: Anada *et al*. recently showed a pro-osteogenic effect of octacalcium phosphate in GM^38^. We confirmed the same for HAp-containing GM-bioinks^39^. In terms of vascularization, Anada *et al*. showed increased vascular sprouting at reduced biopolymer concentration in GM hydrogels^38^. Chen *et al*. observed similar effects upon reduction of the number of methacrylic functions in GM. Obviously the composition of GM hydrogels is crucial to support specific functions of specified cells.

GM can be provided with various amounts of methacryl groups. However, changes in the degree of methacryloylation of GM result in interconnected effects on the solution viscosity as well as on the stiffness and swellability of the crosslinked material^40^. The same is true upon variation of the biopolymer concentration: Decreasing the GM concentration will reduce the density of crosslinks within the hydrogel. Consequently, the swellability of the crosslinked hydrogel will increase and the storage modulus will go down, while at the same time the viscosity of the hydrogel precursor solution will also drop. Considering the mutual dependence of solution properties and hydrogel properties in GM systems, it becomes clear that simultaneous adjustment of rheological, mechanical, and biological hydrogel properties is not always possible. Blends with polysaccharides^41,42^ or with un-modified gelatin^43^ have been applied to compensate for insufficient viscosity in GM based solutions for extrusion-based bioprinting. In earlier works we have already introduced an additional GM-based component, acetylated GM (GMA)^28,29,44–46^. GMA adds additional degrees of freedom to the formulation of all gelatin-based bioinks and hydrogel scaffolds. The acetyl-modification of GM, which is achieved by additional transformation of amino- and hydroxyl groups into acetyl residues, reduces the capability of GMA to form hydrogen bridges. Therefore, GMA forms solutions with low viscosities^47^. Furthermore, acetyl functions are not involved in the crosslinking reaction and thus GMA leads to hydrogels with less crosslinks than pure GM at equal biopolymer concentrations^28^.

In this study, we aimed to re-formulate a GM hydrogel, which we have applied to promote vascular network formation before^48^, to subsequently adjust it for processing by extrusion-based printing. We want to demonstrate how to achieve printability in extrusion-based bioprinting *and* soft hydrogel scaffolds that promote the formation, maturation and maintenance of vascular-like structures by simply blending the gelatin-based toolbox of G, GM and GMA. The three gelatin-based components have different impacts on the viscosity and the physical (thermal) gel formation of solutions and on the properties of the photo-crosslinked hydrogels:

G shows strong physical gelation, high gelation/melting temperatures, relatively high viscosities, but no chemical crosslinking ability. GM shows reduced viscosities, reduced abilities to form physical gels, and low gelation temperatures in relation to non-modified G. The extent of the decrease depends of the degree of modification of the biopolymer. On the other hand, GM provides methacryl-functions that can be covalently crosslinked upon radical activation. GMA also provides methacrylic -functions and additional acetyl-functions. The resulting increase of the overall degree of modification and steric effects lead to further decrease in viscosity and physical gelation and also reduce the effectivity of chemical crosslinking, yielding softer gels^45,46^. Sophisticated blending of G, GM and GMA may therefore be used to tailor the properties of bioinks and hydrogels.

Finally, we set up a vascularized bone model via extrusion-based printing of a bone bioink and the newly formulated vascularization bioink. We confirm the improved vasculogenic processes through the co-culture with the engineered osteogenic tissue compartment in the bioprinted construct.

## Results and Discussion

**Figure 1:**
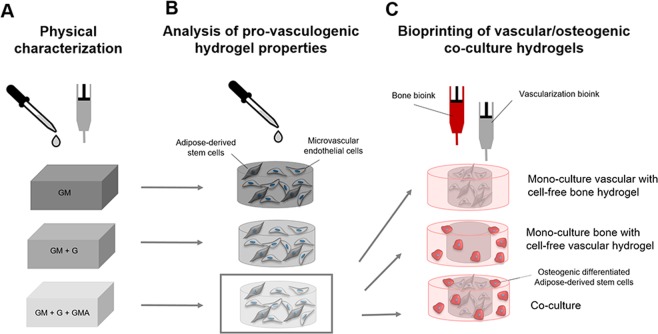
Schematic overview of samples and experimental investigations of the study. (**A**) Manual and extrusion-based preparation of cell-free hydrogels based on GM, GM + G or GM + G + GMA for physical characterization (**B**) Manual preparation of vascularization hydrogels including HDMECs and ASCs (**C**) Extrusion-based preparation of co-culture constructs with a bone compartment including ASCs in bone bioink, and a vascularization compartment including HDMECs and ASCs in vascularization bioink, and respective mono-cultures including either the cell-loaded bone-hydrogel or the cell-loaded vascularization-hydrogel and the cell-free counterpart.

The evaluation of different GM-based formulations was performed in three steps. First, cell-free inks were characterized physically (Fig. 1A). Next, human dermal microvascular endothelial cells (HDMECs) and adipose-derived stem cells (ASCs) were encapsulated to evaluate the resulting hydrogels´ impacts on the formation of vascular structures (Fig. 1B). Finally, the most promising vascularization bioink was selected and used with ASCs and HDMECs to set up 2-phase co-culture constructs with an ASC-containing bone bioink via extrusion-based bioprinting (Fig. 1C)^49^.

We started with solutions and gels of pure 5.75 wt% GM from earlier works^48^, which were tailored to support vasculogenesis, yet have not been adjusted for extrusion bioprinting at 21–22 °C processing temperature. The extruded hydrogel structures have to be physically stable at this temperature (controlled laboratory temperature) until the light-induced chemical crosslinking of the methacryl-functions of GM (and GMA). We changed the formulation by supplementing 1.5–3 wt% unmodified gelatin (GM + G) and by additional replacement of 1.25 wt% GM by 1.25 wt% GMA (GM + G + GMA). We applied a sophisticated trial-and error-approach, which was based on our own knowledge on the gelatin-based materials and on that of others^22,28,45,46^. The viscosity and thermal gelation of the initial formulation of the vascularization ink containing 5.75% (w/w) GM was too low to achieve stable hydrogel structures after extrusion at 20–22 °C. The chemical modification of GM reduces both the viscosity and the physical (thermal) gelation of the biopolymer in solutions. Hence, we expected that the addition of non-modified G will stabilize the GM solution (bioink) directly after printing and before the chemical crosslinking of the methacryl-functions of GM through formation of physical gels. Finally, the non-modified G will be removed from the gel in aqueous environments because it cannot be chemically crosslinked.

In contrast, GMA has relatively lower viscosities than GM, lower thermal gelation temperatures, and a lower capacity of chemical crosslinking. Such characteristics are assigned to steric effects of the additionally inserted, non-reactive acetyl-functions at constant amounts of crosslinkable methacryl-functions^45,46^. Consequently, our hypothesis was that partly exchange of GM with GMA could counteract to a potential increase of viscosity during the actual extrusion process without changing the final biopolymer concentration of the hydrogel.

### Modification of GM-hydrogels with gelatin and acetylated GM improves material properties and printability

**Figure 2:**
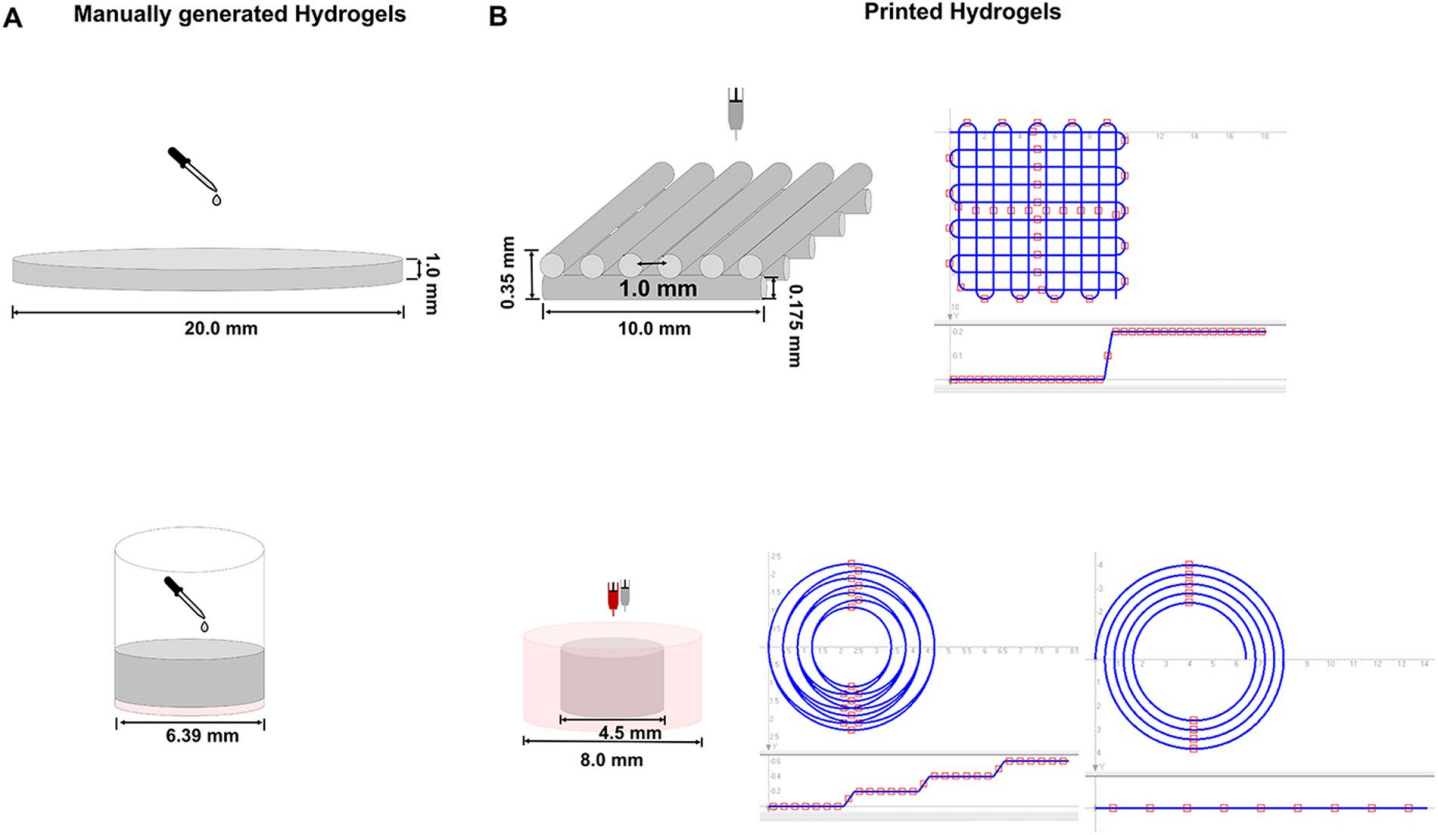
Graphical illustration of manually generated and printed 3D scaffolds. (**A**) Top: Graphical illustration of manual generation of cell-free hydrogels for rheological characterization of storage and loss modulus and gravimetric determination of swellability: The bioink formulations were pipetted into flat cylindrical molds, covered using quartz glass, and cured using 365 nm. Bottom: Graphical illustration of manual generation of the 2-phase hydrogels: Cell-free bone bioink was pipetted into 96-well plate wells (red) and covered with vascularization bioink including HDMECs and ASCs (grey). (**B**) Top: Graphical illustration of printed hydrogels: Cell-free two-layered grid structure for the evaluation of the inks’ printability and trajectory patterns for printing the two-layer grid structure. Bottom: Graphical illustration of printed concentrical co-culture constructs consisting of cell-loaded or cell-free bone bioink (outer part, red) and cell-loaded or cell-free vascularization bioink (inner part, grey) and trajectory patterns for printing the concentric cylinders. (Partly reproduced from^49^).

**Figure 3:**
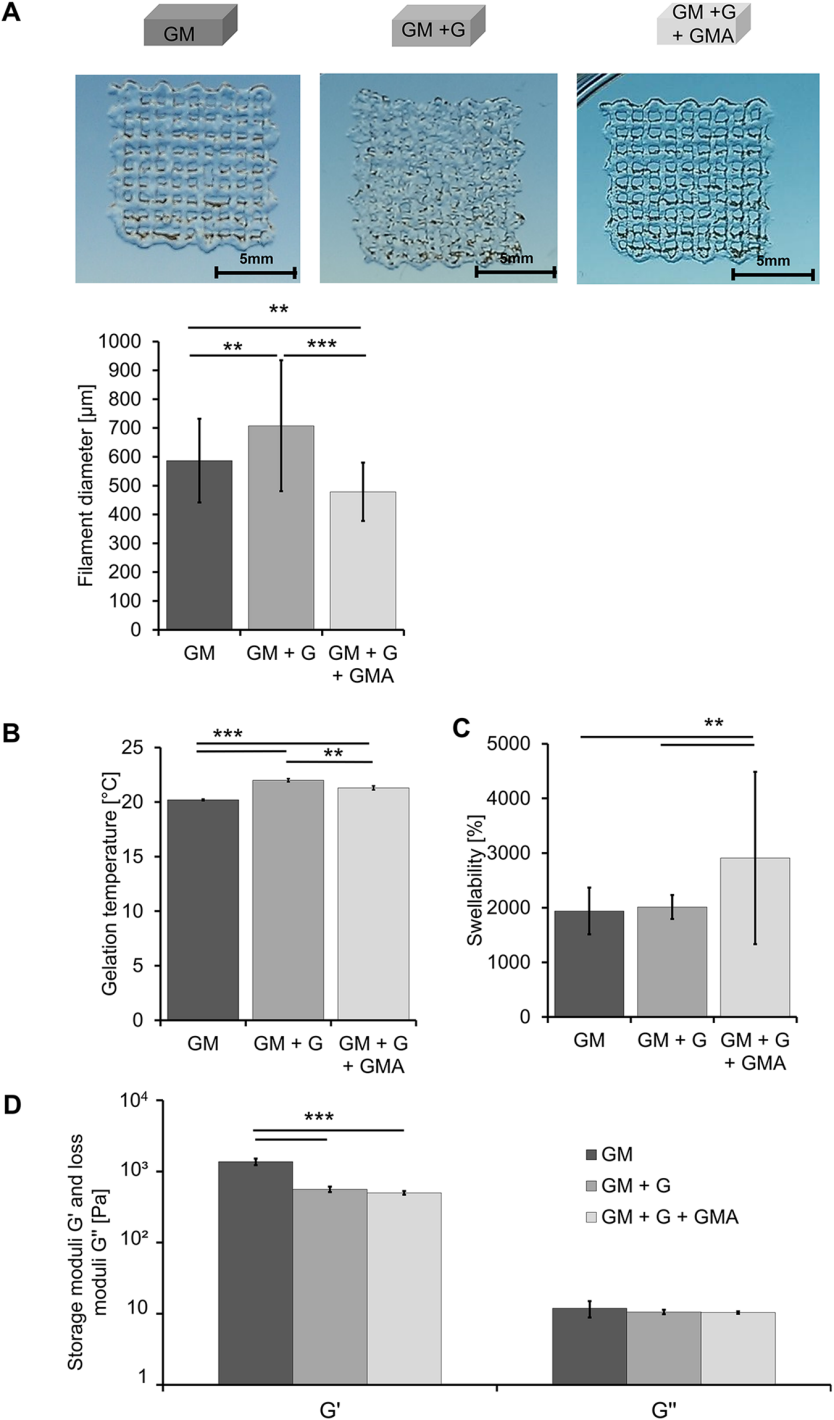
Physical characterization of cell-free bioink hydrogels based on GM, GM + G or GM + G + GMA. (**A**) Extrusion-printing of grid structures and diameters of the resulting filaments. (**B**) Gelation temperatures of the three ink variants in °C. (**C**) Equilibrium degree of swelling of the different hydrogel variants. (**D**) Rheological characterization of the three hydrogel variants. Shown are the storage modulus G′ and the loss modulus G″ in Pa. All results are displayed as mean of three independent measurements and the respective standard deviations. Statistically different means are marked (*p < 0.05; **p < 0.01; ***p < 0.001). (Partly reproduced from^49^).

To assess the extrudability of the inks, a grid geometry consisting of ink filaments arranged orthogonally to each other in two layers was printed (Figs. 2B and 3A).

Pure 5.75 wt% GM-ink resulted in poorly defined filaments, which tended to spread at the fixed processing temperature (21–22 °C). In contrast, the addition of 1.5–3 wt% G yielded an ink that was excessively gelled and the printed filaments were inhomogeneous. Finally, the replacement of 1.25 wt% GM by GMA gave a smooth and excellently extrudable ink, which could be used to print homogenous and well-defined filaments. Pure GM resulted in filaments of a larger diameter (586.7 µm ± 145.17 µm) compared to GM + G + GMA (478.6 µm ± 100.98 µm), which confirms the visual impression. Printed filaments of GM + G showed the highest diameter. The observed inhomogeneity is reflected in the large standard deviation.

The gelling point of a bioink describes the temperature at which the bioink turns from the liquid (storage modulus G′ < loss modulus G″) into a solid (G′ > G″) state and is considered to be the most critical parameter in terms of the ink’s extrudability. The gelation points were 20.2 °C ± 0.07 °C for the pure GM-ink, and significantly higher 22.0 °C ± 0.14 °C and 21.3 °C ± 0.19 °C for the inks modified with G or G and GMA, respectively (Fig. 3B). Obviously, the addition of G not only led to an increase in biopolymer concentration but also to stronger physical gelation. The resulting gelation temperature was raised above the processing temperature and consequently spreading of the ink no longer occurred.

GMA has a high total degree of modification due to additional acetylation of amino- and hydroxy-functions of gelatin and thus a strongly reduced capability to form physical gels. Partial exchange of GM with GMA resulted in reduced inter-molecular interactions and in a gelation temperature that exactly matched the processing temperature and hence enhanced extrudability at room temperature (RT, 21–22 °C)^50^.

The equilibrium degrees of swelling of the hydrogels after UVA-induced crosslinking were determined to obtain the hydrogels’ water uptake capacity, which is assumed to be relevant in terms of vascularization. The swellability of pure GM gels (1) and GM + G gels (2) was 1,939.1% ± 427.07% and 2,012.9% ± 218.90%, respectively. The equilibrium degree of swelling of GM + G + GMA hydrogels (3), however, was significantly higher at 2,910.5% ± 1,576.84% (Fig. 3C).

The storage modulus G′ and the loss modulus G″ were determined for the characterization of the viscoelastic properties of the crosslinked hydrogels (Fig. 3D). The storage modulus G′ was 1,373.21 Pa ± 45.75 Pa for the pure GM-gels; addition of G or G and GMA resulted in significantly lower values of 556.20 Pa ± 5.05 Pa and 500.25 Pa ± 130.66 Pa, respectively.

The low storage moduli of the hydrogel variations (2) and (3) indicate that the presence of G led to steric hindrance of covalent crosslink formation, such that less elastic forces occurred in (2) and (3) compared to (1)^51^. On the other hand, the swellability of the hydrogels remained constant upon addition of G. This suggests that G was physically entangled and at least partly persisted within the hydrogel, thereby contributing to the dry mass of the gel. Hence, the effects of less crosslinks, elevated osmotic concentration and additional solid contents approximately compensated each other in terms of swelling of the hydrogels. The exchange of GM with GMA led to a significant increase of the swellability, which e.g. points to significantly lower density of crosslinks in (3) than in (2) at preserved osmotic concentration within the gel.

The loss moduli of the three hydrogel variants did not show significant differences.

Various studies revealed that weakly crosslinked GM hydrogels, i.e. low degrees of methacryloylation or low concentrations of GM, yielded higher cell viability in general and particularly more effective capillary formation than strongly crosslinked hydrogels^38,43,52^. Thus, the aim is to provide gelatin-based formulations with sufficiently high viscosity or physical gelation to be stable during extrusion-based assembly, and low density of chemical bonds after crosslinking. The latter limits the applicable concentration of GM.

Yin *et al*. used 5% (w/v) of GM and added 8% (w/v) of unmodified gelatin to achieve stable hydrogel extrusion at processing temperatures up to 25 °C. The addition of G further resulted in elevated storage and compression moduli and in reduced swelling, thereby indicating that G was at least partly entangled into the biopolymer network. Targeted reduction of crosslinking density can only be achieved by reducing the concentration of crosslinkable GM in such two-component-systems. This will enhance the water uptake capacity of the gel, but simultaneously the elastic strength will decrease and handling of the gels will become difficult.

In our approach, we also used unmodified gelatin to improve the print-stability of GM-formulations. Furthermore, we provide GMA as additional gelatin-based component. GMA can be used to formulate bioinks with very low viscosities, e.g. for inkjet printing, due to its lack in physical gelation^28^. It can also be used to tune the properties of the crosslinked gels by reducing the number of crosslinks at preserved biopolymer concentration: GMA is covalently integrated into the biopolymer network due to the presence of methacrylic groups. However, masking of amino- and hydroxy groups by acetylation results in less effective crosslinking due to sterical hindrance and impeded physical interactions^44^.

Similar results may be expected by adjusting the degree of methacrylation for the GM component. However, it has to be noted that lower degrees of modification lead to stronger physical gel formation if the processing temperature is below the gelation temperature. This is associated with helix-formation of G and GM molecules, more effective crosslinking and thus stiffer gels^44^. Consequently, the synthesis and handling of tailored GM derivatives requires much experience.

With view to commercial products, the presented multi-component toolbox of G, GM and GMA shows clear advantages, since it allows for flexible formulation of bioinks by blending instead of changing the chemical nature of the single component.

### Modification of GM-hydrogels with gelatin and acetylated GM improves formation of capillary-like structures by encapsulated and supporting cells

**Figure 4:**
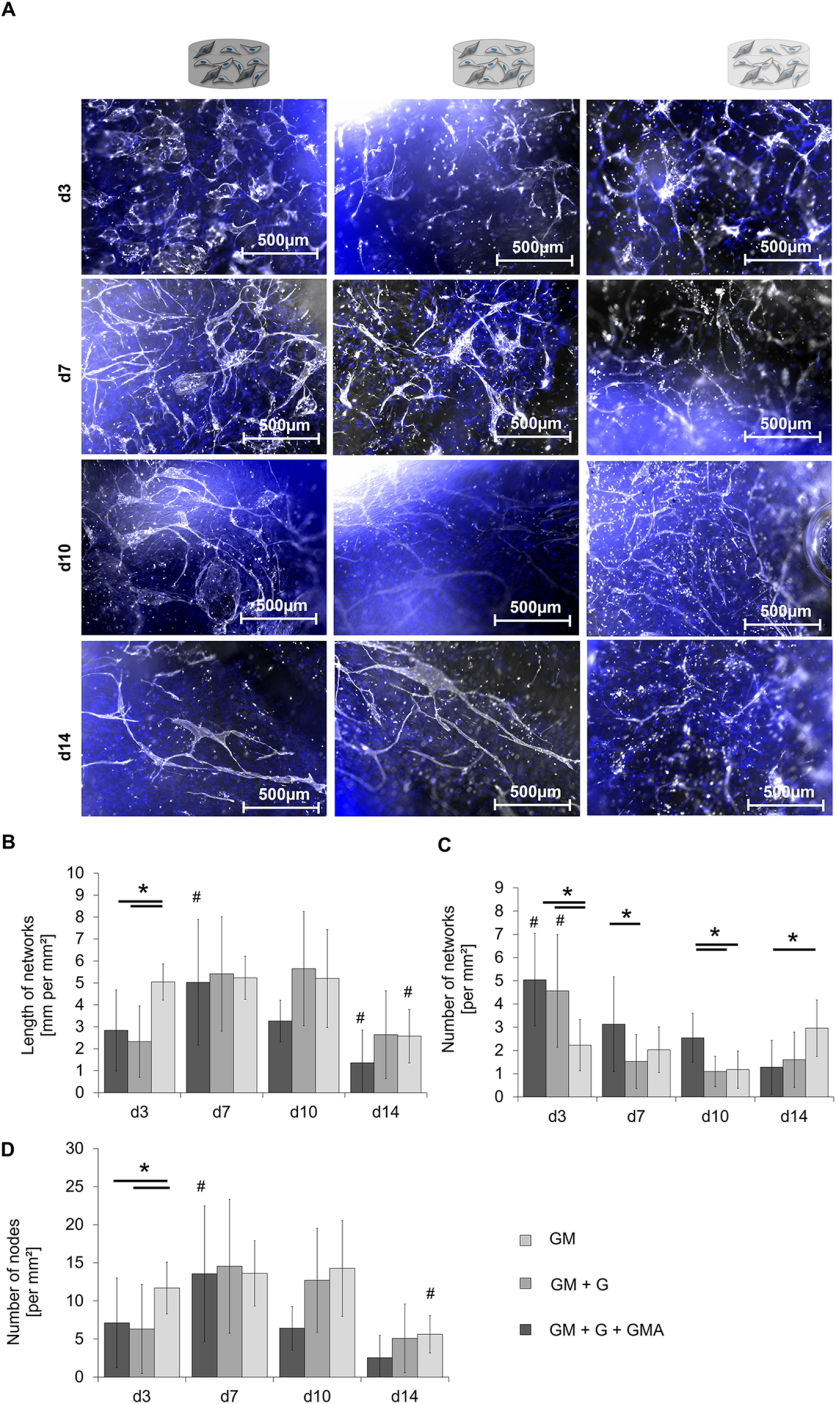
Qualitative and quantitative analysis of capillary-like structures in different hydrogel variants based on GM, GM + G or GM + G + GMA in ASCs-HDMECs co-culture. (**A**) Staining of PECAM-1 in three hydrogel variants on day 3, day 7, day 10, and day 14 of culture. PECAM-1 is shown in white, the DNA in blue. Scale bar 500 µm. (**B**) Total length of the formed network in mm per mm². (**C**) Number of networks per mm² and (**D**) number of nodes per mm² in the formed networks. Shown are the mean values from three independent experiments and the respective standard deviations. Statistically different means (p < 0.05) are marked as follows: # significantly different to all analysis days of the same hydrogel variant; *significantly different between the two conjoined hydrogel variants. (Partly reproduced from^49^).

In vascularization attempts, fibrin is the most frequently used material to support the formation of capillary-like structures^53^. Further studies show that collagen – the basic constituent of gelatin – equally allows for matrix degradation by ECs, EC activation and capillary formation, which are all known to be important steps within vasculogenic and angiogenic processes^54^. We aimed to develop printable gelatin-based matrices to support vasculogenic processes by HDMECs and ASCs. As part of this effort, capillary formation in the different hydrogel formulations was assessed via immunofluorescence (IF) staining of the EC-specific marker platelet endothelial cell adhesion molecule-1 (PECAM-1). Representative pictures of the stained structures on days 3, 7, 10 and 14 of culture are compiled in Fig. 4A. In all three hydrogel variants, capillary-like multicellular structures characterized by the expression of PECAM-1 were already visible at day 3. In a general trend, more and longer capillary-like structures developed and the linking-up between the single structures got more pronounced from day 7 to day 10. On day 14, however, less capillary-like structures were visible and only isolated multicellular aggregates were left.

The networks in GM + G + GMA already exhibited the average length of 5.0 ± 0.8 mm per mm² on day 3, which was significantly higher compared to GM and GM + G. This level was maintained in GM + G + GMA until day 10. Like the other hydrogel compositions, the GM + G + GMA-gels showed a significantly decreased network length of 2.6 ± 1.2 mm per mm^2^ on day 14. Both GM- and GM + G-gels showed significantly higher numbers of networks right from the beginning on day 3 (4–5 networks per mm²) compared to GM + G + GMA (2–3 networks per mm², Fig. 4C). However, the numbers remarkably decreased in GM and GM + G hydrogels until day 14. In contrast, in GM + G + GMA-hydrogels the number increased to an average level of 3.0 ± 1.2 on day 14, which significantly differed from the number obtained in GM. It is noticeable, that both GM and GM + G showed a higher number of networks combined with a lower total structure length, especially at the beginning of the culture period. Therefore, it has to be assumed, that cells in GM + G + GMA hydrogels formed a more crosslinked and therefore more mature network right from the beginning. Although GMA + G + GM suffered from a decrease in the average network length on day 14, the number of networks increased compared to day 10, which might indicate ongoing developmental processes.

The identified total number of nodes (Fig. 4D) is in good correlation with the ratio of length and number of capillary-like structures. It increased and then decreased over time, accounting for fusion and later fragmentation of the present structures. GM + G + GMA showed a significantly higher number of nodes on day 3 with (11.7 ± 3.4 nodes per mm²) than GM (7.1 ± 5.9) and GM + G (6.3 ± 5.8). This additionally supports the hypothesis of enhanced and earlier network formation and associated network maturation in GM + G + GMA formulations compared to the other hydrogels.

The observed positive effects of the hydrogels on the formation of capillary-like structures might be explained by the softer properties of the modified hydrogel compositions compared to pure GM gels as shown in the mechanical characterization of the different hydrogels. A correlation between the elastic modulus of hydrogels and the formation of vascular structures has already been revealed before by Shamloo *et al*. and Chen *et al*.^52,55^. The proposed lower density of crosslinks and thus increased mesh width most presumably led to a simplified migration of the two encapsulated cell-types.

Based on these results, the GM + G + GMA-hydrogel (3) was selected as the most appropriate composition for the formation and maintenance of vascular-like structures, and for extrusion-printing at RT. It was used in subsequent printing experiments for the setup of the vascular compartment in a 2-phase construct for osteogenesis and vasculogenesis.

### GM-hydrogels with gelatin and acetylated GM can be used to build up hydrogels for the co-culture of vascular and osteogenic cells via bioprinting

In a final attempt, the applicability of the selected vascularization ink (3) was evaluated in a co-culture setup, which was assembled via extrusion-based bioprinting (Fig. 2B). The co-culture construct combined osteoblasts in bone bioink, and HDMECs and ASCs in vascularization ink. Reference constructs comprised either a cell-free bone compartment or a cell-free vascular compartment (Fig. 1C). The constructs were cultured for 30 days in co-culture medium.

**Figure 5:**
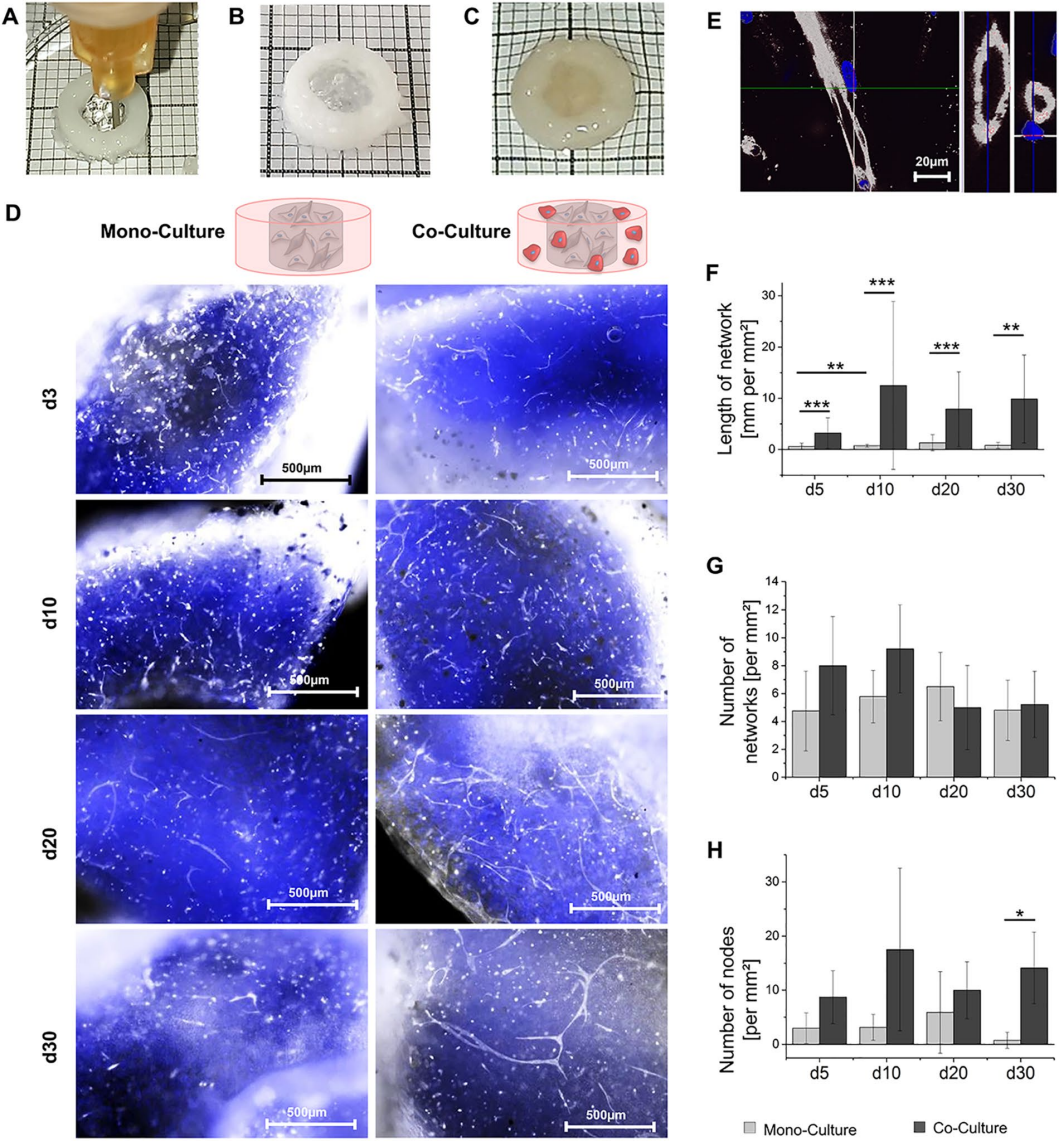
Qualitative and quantitative analysis of capillary-like structures in printed constructs consisting of a core made of vascularization hydrogels of GM2 + G + GMA (3) including HDMECs and ASCs either in a ring made of cell-free bone hydrogels (“mono-culture”) or in a ring made of ASC-laden bone-hydrogels (“co-culture”). (**A**) Macroscopic image of printing process. (**B**) Macroscopic image of printed 2-phase construct. (**C**) Macroscopic image of hydrated, printed 2-phase construct. (**D**) Staining of PECAM-1 on day 5, 10, 20 and 30; PECAM-1 in white, DNA in blue, scale bar 500 µm (**E**) Exemplary lumen-containing structures of capillary-like networks on day 10 in the co-culture setup, displayed in lateral view, diagonal cut and cross section, PECAM-1 in white, DNA in blue, scale bar 20 µm. (**F**) Total length of the formed network in mm per mm² in mono-culture vs. co-culture. (**G**) Number of networks per mm² in mono-culture vs. co-culture and (**H**) number of nodes per mm² in the formed networks in mono-culture vs. co-culture. All results are displayed as mean of three independent measurements and the respective standard deviations. Statistically different means are marked (*p < 0.05; **p < 0.01; ***p < 0.001). (Partly reproduced from^49^).

Both the co-culture approaches and the reference without osteoblasts led to the formation of capillary-like structures, as visualized via IF staining against PECAM-1 in Fig. 5D. In the co-culture setup, it was possible to detect lumen formation by ECs (Fig. 5E). The formed luminal structures showed comparable diameters to those in available literature^56^. Clear differences between the control and co-culture were revealed in the quantitative analysis of vascular formation processes. The control culture started with the observable total network length of 0.6 mm per mm² ± 0.6 mm per mm² on day 5 and did not exceed 0.8 mm per mm² ± 0.3 mm per mm² during the entire culture period (Fig. 5F). In contrast, the total network length in co-culture showed moderate values right from the beginning with 3.2 mm per mm² ± 3.0 mm per mm² and increased to 12.5 mm per mm² ± 16.4 mm per mm² on day 10, with no significant decline until day 30. Significantly more nodes were preserved in co-culture constructs after 30 days of culture with 14.1 ± 6.6 nodes per mm² in the co- and 0.8 ± 1.5 nodes per mm² in control culture (Fig. 5H). The obtained data suggests a faster and more pronounced formation of vascular structures under co-culture conditions. No remodeling was observed after the initial formation until day 10, and the formed structures were maintained until day 30.

**Figure 6:**
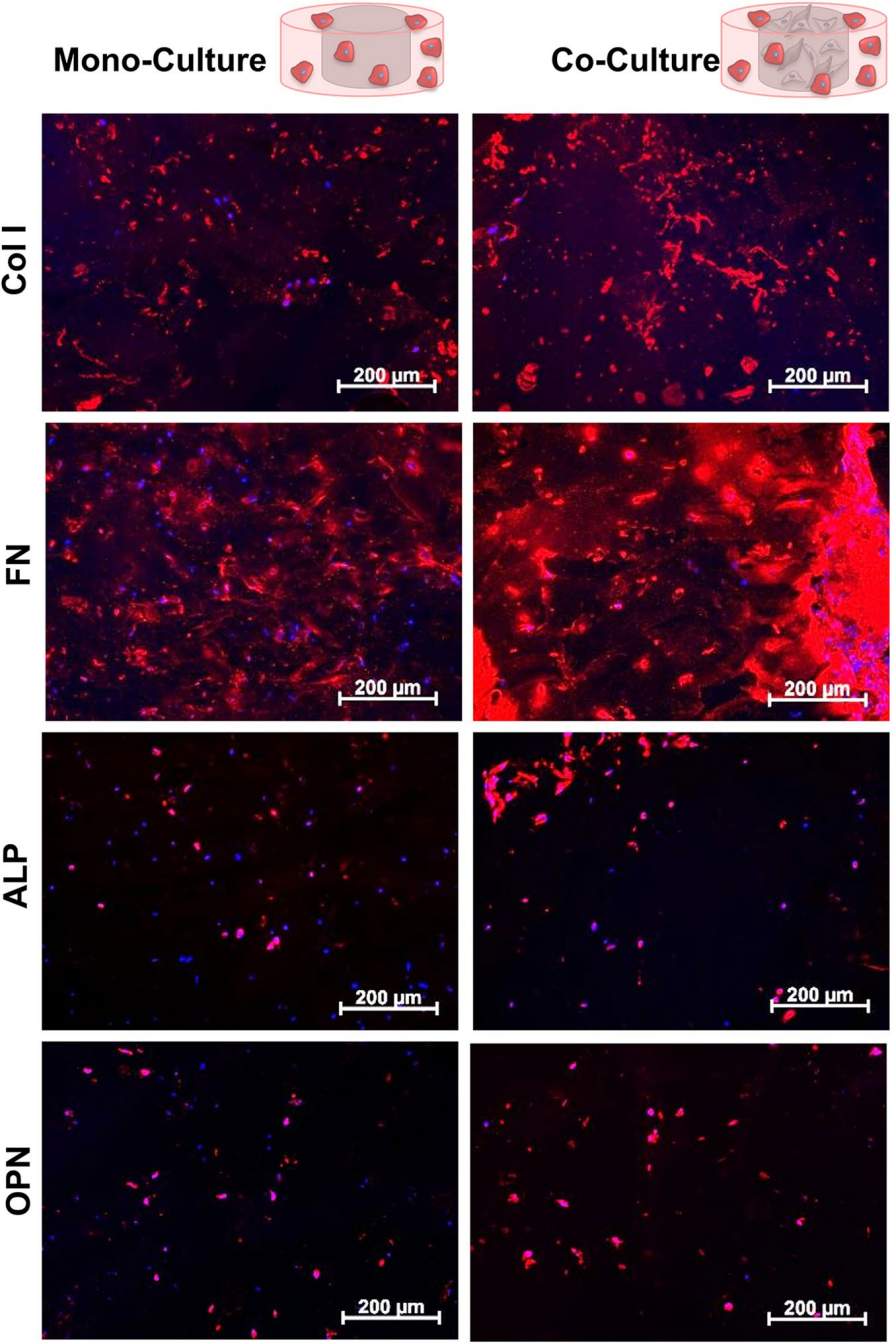
Expression of bone-associated matrix proteins in printed concentric constructs consisting of a ring made of ASC-loaded bone-hydrogels either with a core made of cell-free vascularization hydrogels (“mono culture”) or a core including HDMECs and ASCs (“co-culture”). Exemplary pictures of mono- and co-culture on day 20, Col I, FN, ALP and OPN are displayed in red, DNA in blue, scale bar 200 µm. (Partly reproduced from^49^).

In addition to the formation of vascular-like structures, the deposition of bone-matrix-specific proteins was analyzed via an IF staining of collagen type I (Col I), alkaline phosphatase (ALP), osteopontin (OPN), and fibronectin (FN), (Fig. 6). Expression of such proteins indicate the differentiation of ASCs to osteoblasts. While almost no expression of Col I or FN was visible on day 1 (Supplementary Fig. S1), a prominent staining was obtained on day 10. Based on the visual evaluation of the IF images, the co-culture resulted in a more pronounced expression of the proteins especially on day 20 (Fig. 6). ALP was only expressed in perinuclear regions on day 1 and showed extracellular expression starting on day 10. However, no clear differences became obvious between the co-culture constructs and the reference without vascular cells. In contrast, OPN expression increased in co-culture on day 10, while in the control culture it stayed at a low level throughout the whole period.

The new gelatin-based formulation was established as bioink for automated deposition of vascular cells. It was combined with another cell-type specific bioink formulation in an extrusion-based bioprinting process and it enabled the formation of crosslinked 3D hydrogel scaffolds, which supported the generation of vascular structures.

The formation of bone matrix and the formation of vascular structures are interdependent processes *in vivo*^13,57–60^. The current study confirms published data on this reciprocal influence *in vitro* supporting not only the formation of vascular structures, but also the deposition of specific bone matrix proteins. While most previous attempts based their evaluation on 2D culture systems, our current approach confirmed the crosstalk between osteogenic and vasculogenic cells towards the formation of vascularized BT *in vitro* in a 3D culture setup established by extrusion-based bioprinting. Due to the co-culture in two different hydrogel compartments with only limited possibility for direct cell-cell contact, the obtained effects mainly have to be assigned to paracrine communication between the different cell types.

Vascular processes, e.g. expression of receptors for VEGF on ECs, are known to be upregulated after paracrine communication with osteogenic cells^60^. In combination with VEGF, secreted from osteoblasts, this could have led to a support of vasculogenic processes.

The remarkably reduced degree of vascularization in the reference cultures as shown in Fig. 5 (comparison of printed co-culture and reference culture without osteoblasts) relative to the mono-cultures as shown in Fig. 4 (comparison of different hydrogel formulations) presumably results from different compositions of the used culture media: The cell culture medium in the co-culture experiments with 2-phase hydrogels contained reduced concentrations of VEGF and basic fibroblast growth factor (bFGF), both known to be supporting factors of vasculogenic processes. This deficiency was apparently compensated in co-culture by paracrine factors of osteogenic cells but not in mono-culture of the vascular cells without osteoblasts.

Earlier co-culture studies with ECs by Kaigler *et al*., Grellier *et al*., Villars *et al*. and Wang *et al*. already showed increased expressions of proteins associated with osteogenic differentiation like CI, ALP and OPN^59–62^. The induction of these osteogenic proteins was shown to be linked to the release of bone morphogenetic protein 2 by ECs in response to VEGF^16^.

## Summary and Conclusion

In the current study, we successfully modified our existing GM-based hydrogel formulation for 3D *in vitro* vascularization by the addition of non-modified gelatin (G) and acetylated GM (GMA), and achieved improved printability with extrusion-based printing systems. The resulting hydrogels showed improved material properties after crosslinking, i.e. reduced crosslinking density and elevated swelling, which are assumed to be important for capillary formation and maintenance. The optimized bioinks allowed the bioprinting of co-culture hydrogels including HDMECs and ASCs for the vascular compartment, and ASCs for the osteogenic compartment. The integrated cells stayed viable throughout the printing process. Cellular crosstalk led to further differentiation of the ASCs in the osteogenic compartment, which was observed by expression of bone-specific proteins Col I, OPN and FN, and a self-guided, stabilized assembly of capillary-like networks in the vascular compartment.

Bioprinting will provide the technical base for automated and reproducible production of biological tissue for *in vitro* tests or as biological implants of the future. This study contributes to the development of bioprinting by the presented bioink formulation, which matches the process requirements for automated, extrusion-based assembly as well as biological requirements to support formation of capillaries.

The presented ink is purely composed of G and G derivatives and can be remodeled and degraded by cells. It can be combined with other bioink formulations that we described earlier and that support the osteogenic differentiation of ASCs^32,39,48^.

With view to commercial products, we offer this multi-component toolbox of G, GM and GMA that allows for flexible formulation of bioinks by blending instead of changing the chemical nature of the single component.

## Methods

### Cell isolation and culture

HDMECs and ASCs were isolated from human skin biopsies from plastic surgeries (Klinik Charlottenhaus, Stuttgart). All research was carried out in accordance with the rules for investigation of human subjects as defined in the Declaration of Helsinki. Patients gave written informed consent according to the approval of the Landesärztekammer Baden-Württemberg (F-2012–078; for normal skin from elective surgeries).

HDMECs were isolated from the dermis of three female donors aged 35 to 54 as described by Huber *et al*.^63^. The cells were squeezed in pre-warmed endothelial growth medium (EGM)−2 (Lonza, Switzerland) with a scalpel, cultured in EGM-2 and subcultured until passage 2.

Human ASCs were isolated from subcutaneous adipose tissue according to Huber *et al*.^63^. The obtained stromal vascular fraction was suspended in Mesenchymal stem cell growth medium (MSCGM, Lonza, Switzerland) with 2% FBS and expanded on TCPS^32,39^.

### Preparation of vascularization bioinks

In this study, three different bioink compositions based on GM, G and GMA were prepared and evaluated regarding their potential to support the formation of capillaries by HDMECs and ASCs, and regarding their suitability for extrusion-based bioprinting. The production of GM_2_ (in this study named GM) and GM_2_A_8_ (in this study named GMA) is described in^32,39^, with the subscript numbers indicating the mode of methacrylation or acetylation, respectively. The average degrees of modification are 0.32 mmol g^−1^ methacryloyl per GM_2_, 0.38 mmol g^−1^ methacryloyl per GM_2_A_8_, 0.4 mmol g^−1^ acetyl per GM_2_A_8_.The following formulations of bioinks were prepared by solubilizing the biopolymers in phosphate buffered saline (PBS) at 60 °C for at least 60 min.

1. GM_2_ [5.75 wt%]
2. GM_2_ [5.75 wt%] + G [1.5–3 wt%]
3. GM_2_ [4.5 wt%] + GM_2_A_8_ [1.25 wt%] + G [1.5–3 wt%]

Depending on the exact properties of each GM_2_ lot used, the concentration of G in the ink (hydrogel precursor solution) was adjusted in the range of 1.5–3 wt% to enable proper printing. For the ink characterization experiments, solutions with a gelatin concentration of 1.5 wt% were used, while the cell encapsulation experiments were done using hydrogel precursor solution with 3 wt% gelatin.

The photo-initiator lithium phenyl-2,4,6-trimethylbenzoylphosphinate (LAP, synthesized as described in^51^) was added to the solutions at a concentration of 0.2% w/w of the biopolymer mass to generate stable hydrogels by photo-induced crosslinking of the bioinks^48^.

Cells were re-suspended in pre-warmed bioinks using 3 × 10^6^ HDMECs and 3 × 10^6^ ASCs per mL ink (Fig. 1B) to achieve a cell-loaded vascularization bioink. Before ink preparation, the dry GM-, GMA- and gelatin materials were heated to 60 °C under vacuum for at least 24 h for disinfection, or the prepared inks were alternatively sterilized by filtration (cellulose acetate membrane; pore size = 0.2 µm; Sartorius Stedim, Germany).

### Preparation of bone bioink

We used the bone bioink formulation as we introduced it before^32^. Cell-free bone bioink was prepared by solubilization of 7–8 wt% GM_5_, 4–5 wt% GM_2_, 1 wt% methacryl-modified hyaluronic acid (HAM_5_)^32^ and 0.135% (w/w of the biopolymer mass) LAP in PBS at 60 °C for at least 60 min. Subsequent addition of 5 wt% HAp nanoparticles (Sigma Aldrich, Germany; d = 200 nm), and sonication of the suspension to break down aggregates followed.

Cell-loaded bioink was prepared by careful suspension of ASCs (5 × 10^6^ mL^−1^) into the bone ink.

### Physical characterization of cell-free bioinks and crosslinked hydrogels for vascularization

Bioinks and hydrogels were prepared without cells for the physical characterization (Fig. 1A). For the determination of gelation points, which represent the temperature at sol-to-gel transition, and the viscoelastic gel properties in terms of storage modulus G′ and the loss modulus G″, a Physica Modular Compact MCR301 rheometer (Anton Paar GmbH, Germany) was used.

The gel points of the vascularization bioinks were determined by oscillatory measurements with fixed amplitude γ (0.1%) and frequency f (1.0 Hz). A volume of 1.4 mL of the respective bioink at a temperature of 37 °C was pipetted into the measuring gap (distance cone-plate: 401 µm). The temperature was then varied between 25 °C and 15 °C at a cooling rate of 0.4 °C min^−1^ while storage modulus G′ and loss modulus G″ were taken at 100 measuring points. The gel point was determined at equal G′ and G″, corresponding to tan δ = G″/G′ = 1.

Cell-free hydrogels were prepared by photo-induced radical crosslinking of cell-free vascularization bioinks within cylindrical molds. The photo-initiator LAP was added at a concentration of 0.2% w/w of the biopolymer mass^48^. A volume of 315 µL of bioink was pipetted into each mold (25 mm diameter, 1 mm height), covered with a glass plate, and irradiated using UVA light (365 nm, 0.54 J cm^−^², 60 sec) in a radiation chamber.

The equilibrium degree of swelling of the respective hydrogels was characterized by determination of the mass ratio of stored water and dried gels. The gels were equilibrated in PBS at 37 °C, 5% CO_2_ for 24 h to guarantee complete swelling, gently patted dry with a delicate task wipe, and the mass of the swollen gels was determined with a precision scale (Ohaus Corp., US). Afterwards, the gels were vacuum-dried at 60 °C for 48 h and weighed again to determine the mass of the dried gel. The degree of swelling was calculated as follows:

$$degree\,of\,swelling\,[ \% ]=\frac{mass\,of\,swollen\,gel-mass\,of\,dried\,gel}{mass\,of\,dried\,gel}\times {100}$$

The viscoelastic properties of cell-free hydrogels were characterized by oscillatory strain amplitude sweeps (0.01% ≤ γ ≤ 100%) with the rheometer (Anton Paar, Germany) using parallel plates at 37 °C, a normal force F_N_ = 0.32 N and a frequency f = 1.0 Hz. The storage modulus G′ and the loss modulus G″ were determined from the linear viscoelastic range of the hydrogels.

### Manual preparation of cell-laden hydrogels

For the vascularization and co-culture experiments cells from three different donors were used in independent experiments. To evaluate the hydrogels’ influence on the formation and maintenance of capillary-like structures, HDMECs and supporting ASCs were resuspended into vascularization bioinks (1), (2) and (3), as described above (“Preparation of vascularization bioinks”).

96-well plates were precoated with 15 µL of a bone ink (4.5 wt% GM2 + 8.5 wt% GM5 + 0.5 wt% HAM5) which was subsequently polymerized at 0.54 J/cm^2^ for 120 sec to mimic the vascular/bone interface. Following, cell-loaded vascularization inks were pipetted into the precoated wells with 60 µl each and crosslinked in the UVA crosslinking chamber with 0.54 J/cm² for 60 sec.

Cell-containing vascularization hydrogels were cultured for 14 days in EGM-2 with 50 ng mL^−1^ VEGF and 50 ng mL^−1^ bFGF. Evaluation regarding the expression of PECAM-1 and quantification of vascular-like structure formation took place on days 3, 7, 10 and 14.

### Extrusion-based bioprinting

Extrusion-based printing was performed by a prototype machine constructed on the base of the automated tabletop robot TR300 by Unitechnologies SA (Switzerland). The bioinks were extruded from syringes using stainless steel dispensing tips with inner diameter of 0.33 mm (Vieweg GmbH, Germany). Dispensing was performed in a conditioned laboratory at 21–22 °C. The volume per layer and the volume flow rate varied according to the ink used. The printer was equipped with a LED-UVA lamp (385 nm) for curing the extruded hydrogels. For the bone gels, the layers were cured separately for 60 sec each (0.54 J/cm^2^) to account for the light absorption by the HAp particles; for the transparent vascularization gels, all layers were collectively cured for 60 sec (0.54 J/cm^2^).

#### Printing cell-free bioinks

To evaluate the vascularization inks’ extrudability, two-layered grid structures with an edge length of 10 mm (Fig. 2B) were printed using cell-free vascularization bioink formulations (1), (2) and (3). Each layer comprised a volume of 30 µL and was printed with a volume flow rate of 0.22 µL/s.

#### Printing combinations of cell-loaded bioinks

Constructs consisting of a vascular compartment and an osteogenic compartment were printed to evaluate the suitability of the selected bioink for assembly of vascularized bone constructs via extrusion-based bioprinting (Figs. 1C and 2B).

Ten layers of cell-loaded bone bioink with 10 µL each were placed on the print bed with a volume flow rate of 0.22 µL s^−1^.

Cell-loaded vascularization bioink (3), which was selected as most suitable with regard to its printability, swelling behavior and the support of vasculogenic processes, was printed in four layers using 7.5 µL volume per layer with a volume flow rate of 0.3 µL s^−1^.Models with either a cell-free vascularization compartment or a cell-free bone compartment were produced as controls to the 2-phase, vascularized bone constructs (Fig. 1C). All constructs were cultured for 30 days in a combined osteogenic and vasculogenic co-culture medium, consisting of 50% Dulbecco’s Modified Eagle Medium (DMEM) and 50% EGM-2, supplemented with 5% FBS, 25 µg mL^−1^ L-ascorbic acid, 5 mM β-glycerophosphate, 50 nM dexamethasone, as well as 25 ng mL^−1^ VEGF and bFGF each. Evaluation of osteogenic protein expression and formation of vascular-like structures took place on day 5, 10, 20 and 30.

### Immunofluorescence staining and network evaluation experiments

Gels were fixed in an ethanol/acetone (1:1) mixture for 12 min at −20 °C. For IF staining, the samples were permeabilized with 0.2% Saponin in PBS^+^ for 40 min at RT. Then, a blocking step in 0.1% Triton X-100 and 3% bovine serum albumin in PBS^+^ (blocking solution) for 60 min at RT followed. Subsequently, monoclonal antibodies were applied according to Table 1 in fresh blocking solution and incubated for 16 h at 4 °C. After washing with 0.1% Tween20 in PBS^+^ (PBST), secondary antibodies in combination with 1 µg mL^−1^ 4′,6-Diamidin-2-phenylindol (DAPI) in fresh blocking solution were added for 90 min at RT and protected from light. Finally, the samples were washed with PBST for four times and with Millipore H_2_O once. Evaluation took place on an Axiovert or laser scanning fluorescence microscope (Zeiss, Germany).

**Table 1:** Used Antibodies with host and concentration.

Antibody	Host	Concentration of use
**Primary**		
Alkaline phosphatase	Rabbit	0.976 µg mL^−1^
PECAM-1	Mouse	4.1 mg mL^−1^
Fibronectin	Rabbit	9,8 mg mL^−1^
Collagen type I	Mouse	unknown
Osteopontin	Rabbit	200 µg mL^−1^
**Secondary**		
mouse	goat	3 µg mL^−1^
rabbit	goat	4 µg mL^−1^

### Statistical analysis

EC elongation and network formation were analyzed by IF images showing PECAM-1. Images were taken in the middle of the gel halves and analyzed by using the image analysis software ‘Image J’. The average diameter of printed filaments were measured based on the macroscopic images of the printed grid structures using the software ‘Image J’. Statistical analysis was performed by using the OriginPro software. All data are expressed as means ± standard deviations of the means. Statistical significance was tested at p < 0.05 by using a one-way analysis of variance (ANOVA) and a Fisher’s Least Significant Difference (LSD) post-hoc test.

## Supplementary information

Dataset S1.

## Data Availability

The datasets generated during and/or analyzed during the current study are available from the corresponding author on reasonable request.
