# Supplementary material for: Advanced gelatin-based vascularization bioinks for extrusion-based bioprinting of vascularized bone equivalents
Source: Sci Rep. 2020 Mar 24;10:5330. doi: 10.1038/s41598-020-62166-w (PMC7093518; doi:10.1038/s41598-020-62166-w)
Supplement: Supplementary file 1 — Dataset S1. [file 41598_2020_62166_MOESM1_ESM.docx]

**Advanced gelatin-based vascularization bioinks for extrusion-based bioprinting of vascularized bone equivalents**

**A. Leucht, A-C. Volz, J. Rogal, K. Borchers, and PJ. Kluger**


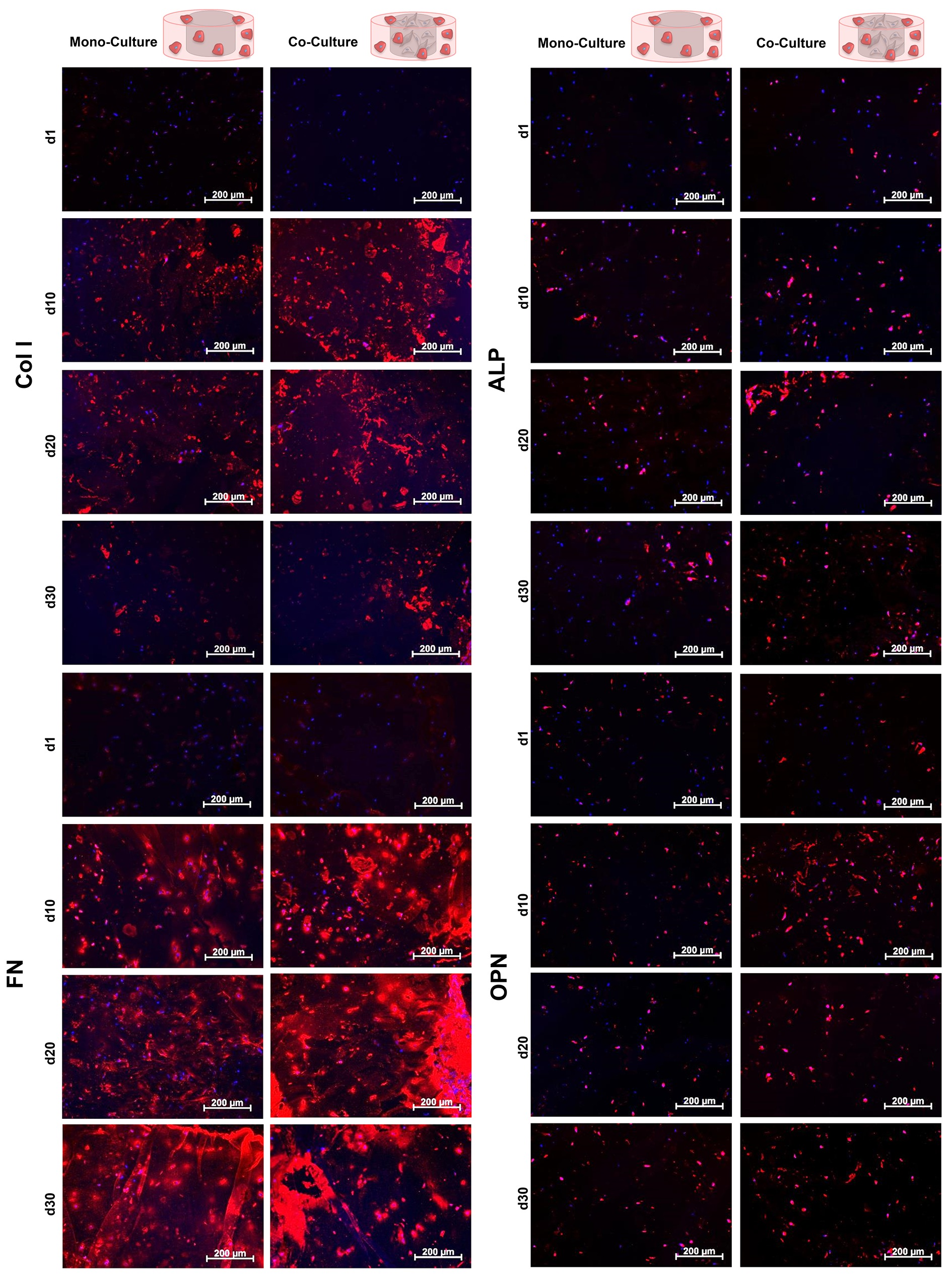
**Figure S1:** Expression of bone-associated matrix proteins in printed constructs consisting of a ring made of ASC-loaded bone-hydrogels either with a core made of cell-free vascularization hydrogels of GM+G+GMA (“control culture”) or a core including HDMECs and ASCs (“co-culture”). Exemplary pictures of control and co-culture on day 5, 10, 20 and 30, Col I, FN, ALP and OPN are displayed in red, DNA I blue, scale bar 200 µm. (Partly reproduced from **^49^**)
